# A High Serum Phosphate and Calcium-Phosphate Product Is Associated With Cerebral Small Vascular Disease in Patients With Stroke: A Real-World Study

**DOI:** 10.3389/fnut.2022.801667

**Published:** 2022-04-04

**Authors:** Wenjing Lv, Can Cui, Zixuan Wang, Junqi Jiang, Binbin Deng

**Affiliations:** ^1^Department of Geriatrics, The Affiliated Hospital of Qingdao University, Qingdao, China; ^2^Institute of Environmental Medicine, Karolinska Institutet, Stockholm, Sweden; ^3^Medical College, Qingdao University, Qingdao, China; ^4^Department of Neurology, First Affiliated Hospital of Wenzhou Medical University, Wenzhou, China

**Keywords:** cerebral small vessel disease, calcium, phosphorus, risk factors, stroke

## Abstract

Cerebral small vessel disease (CSVD) is a slowly progressive disease, often accompanied by stroke, and results in dementia, depression, and cognitive impairment. It was already known that calcium and phosphorus metabolism (CPM) disorders were associated with vascular-related adverse events. The risk factors of CSVD and the relationship between serum calcium (Ca), phosphorus (P), calcium-phosphate product (Ca × P), and CSVD in patients with stroke without CPM disorders are still obscure. In our study, 528 patients with stroke without CPM disorders were enrolled in a cohort from a consecutive hospital-based stroke registry, with 488 patients with CSVD as cases and 140 without CSVD as controls. The patients with CSVD were further sub-grouped into lacunes, white matter hyperintensities (WMHs), and cerebral microbleeds (CMBs). By applying univariate and multivariate logistic regression analysis, the following novel findings were obtained: (i) up to 76.19% of patients with stroke had signs of CSVD, and lacunes are the most common subtype. Notably, 22.96% of patients with CSVD had multiple subtypes coexisted. (ii) Compared with patients without CSVD, patients with CSVD had higher levels of age, rate of hypertension or diabetes, serum Ca, P, Ca × P, and lower levels of white blood cell (WBC) and hemoglobin (HB). (iii) We developed 2 predictive models and nomograms for predicting CSVD, in addition to the known factors (age and hypertension). The levels of P and Ca × P were positively correlated with the risk of CSVD (P: OR = 3,720.401, 95% CI (646.665–21,404.249); Ca × P: OR = 1.294, 95% CI (1.222–1.370)). (iv) The models were further validated in subtypes of CSVD, including lacunes, WMHs, and CMBs, and the results were still valid among the subtypes. In summary, CSVD was highly prevalent in patients with stroke, and high serum P and Ca × P are potential risk factors of CSVD and all subtypes including lacunes, WMHs, and CMBs.

## Highlight

- First, cerebral small vessel disease (CSVD) was first reported to be highly associated with stroke in patients without calcium and phosphorus metabolism (CPM) disorders.
- Second, serum calcium (Ca), phosphorus (P), and Ca × P were found higher in patients with CSVD, than patients without CSVD, in the absence of CPM disorders.
- Third, despite the already known risk factors (age and hypertension), serum P and Ca × P are positively correlated with CSVD, as well as the 3 subtypes of CSVD, including lacunes, white matter hyperintensities (WMHs), and cerebral microbleeds (CMBs), in the absence of CPM disorders.

## Introduction

Cerebral small vessel disease (CSVD) is a prevalent neurological disease in older people, which affects cerebral arterioles, capillaries, and venules. It causes up to 45% of dementia and 20% of all strokes. Besides, cognitive dysfunction, depression, and gait imbalance can be frequently seen in patients with CSVD (1). The diagnosis of CSVD is based on findings of MRI scans including lacunes, white matter hyperintensities (WMHs), and cerebral microbleeds (CMBs) (2). Epidemiology of CSVD indicated discrepancy among the Asian population compared with the non-Asia population (2). In the USA, CSVD was found highly associated with large artery atherosclerosis and often accompanied by stroke (3). It is, however, often undetected when accompanied by stroke among inpatients, probably because the symptoms are less devastating and often progresses slower than those in large vessel occlusion. Since few studies have investigated the epidemiology of CVSD when accompanied by a stroke in China. Antiplatelet treatment and management of traditional risk factors are still considered to be the most important therapeutic and preventive approaches. However, vascular risk factors and large artery disease explain less variance of CSVD (4). Finding the attributable risk factors of CSVD is in great need for preventing and treating CSVD.

As a part of the atherosclerotic process and considered as ectopic deposition of bone components, artery calcification was found highly associated with WMHs, lacunes, and CMBs (5). Serum phosphate (P), calcium (Ca), and calcium-phosphate product (Ca × P) are calcium and phosphorus metabolism (CPM)-related factors. In extreme cases, such as chronic kidney diseases (CKD), disturbance of CPM homeostasis can result in ectopic vascular calcification in blood vessels, as well as elevated P levels and increased Ca × P (6). Lower dialysate Ca concentration can decrease aortic stiffness and carotid intima-media thickness (7). However, less attention has been paid to the potential involvement of CPM disturbance in patients with CSVD without CPM disorders.

## Materials and Methods

### Subjects and Setting

**Figure 1:**
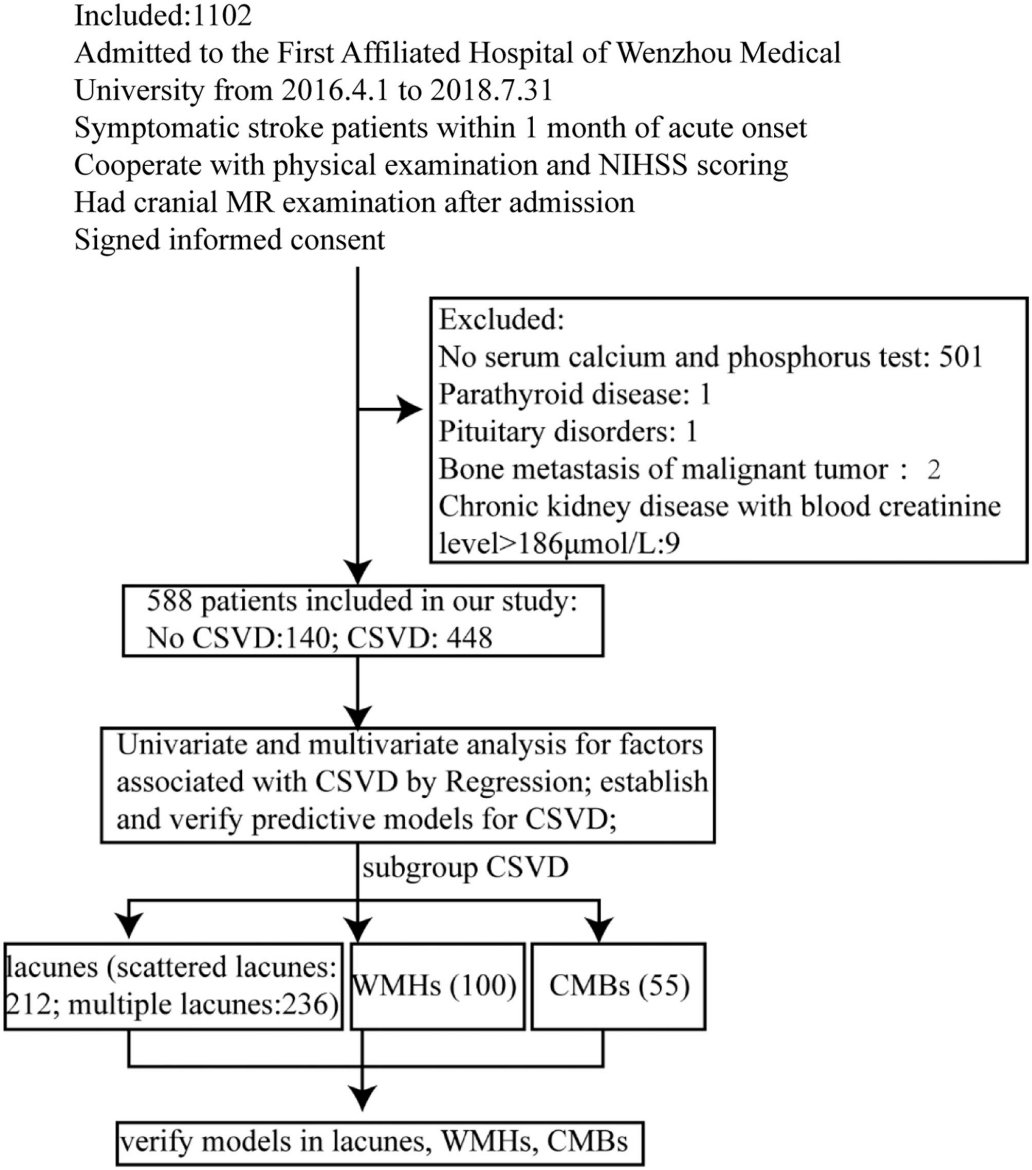
Flow diagram showing the patient selection and data analysis.

From 01 April 2016 to 31 July 2018, there were 1,102 individuals admitted to the First Affiliated Hospital of Wenzhou Medical University. All individuals had to meet the following inclusion criteria: patients with symptomatic stroke within 1 month of acute onset; cooperate with physical examination and National Institutes of Health Neurological Deficit Score (NIHSS) scoring; cranial MR examination after admission; and signed informed consent. The subjects were excluded if they lack serum Ca and P tests, or had any diseases that could influence CPM, including chronic kidney disease with blood creatinine level >186 μmol/L, bone metastasis of malignant tumor, parathyroid disease, and pituitary disorder (Figure 1). After exclusion, 588 patients were included in this study. Among them, 140 patients without any sign of CSVD were categorized as “no CSVD group,” and 448 with any sign of CSVD in the cranial MR images were categorized as “CSVD group.” Within the CSVD group, 212 patients were presented with scattered lacunes, and 236 were presented with multiple lacunes. Notably, 100 of the included cases had WMHs, and 55 of the included cases had CMBs. Cases with overlapping subtypes were repeated included in each subtype.

### Ascertainment of CSVD

Cerebral small vessel diseases are presented with single or combined signs of lacunes, WMHs, and CMBs on MR imaging (2). Lacunes are shown as hypointense lesions with the hyperintense rim on FLARE and T1 and hyperintense signal on T2 in the white matter, basal ganglia, pons, or brainstem. The diameter of the lesions ranges from 3 to15 mm, and the shape of the lesions can be rounded, ovoid, or tubular. Recent small ischemic lacunes are also included in the lacunes identified by hyperintense on DWI, T2, and FLAIR and hypointense on T1, with a usual diameter ≤ 20 mm. WMHs are diagnosed when Fazekas score ≥2. Abnormal WMHs are usually symmetrically and bilaterally distributed in the white matter of the pons and brainstem area and can also occur in deep gray matter. They show patchy or confluent hyperintense signals on T2 or FLAIR. CMBs are defined as small round and homogeneous hypointense foci on T2-weighted MRI and susceptibility-weighted imaging.

### Clinical and Laboratory Assessments

The information of demographics (age, gender, and weight), vascular risk factors (hypertension, diabetes, coronary artery disease, atrial fibrillation, hyperlipidemia, history of smoking, and drinking alcohol), blood biochemistry, vital signs on admission (NIHSS, mean blood pressure (MBP), fasting blood glucose (FBG), 2-h post-meal blood glucose (P2Hpg), glycated HB (HbA1c), total cholesterol (TC), triglyceride (TG), low-density lipoprotein cholesterol (LDL), high-density lipoprotein cholesterol (HDL), left ventricular ejection fraction (LVEF), WBCs, neutrophils, lymphocytes, red blood cells (RBCs), HB, total serum protein (TP), albumin, blood platelet (PLT), total prothrombin time (PT), international normalized ratio (INR), activated partial thromboplastin time (APTT), fibrinogen (FIB), urea nitrogen (BUN), creatinine (Cr), homocysteine (HCY), thyroid-stimulating hormone (TSH), free triiodothyronine (FT3), free tetraiodothyronine (FT4), serum biochemical markers of CPM (P, Ca, adjusted Ca, Ca × P, and adjusted Ca × P), diagnosis, and imaging findings of all patients were collected from standardized data records. All hematological tests were taken on the morning of the second day of admission after overnight fasting. Cranial MR examinations were completed within 3 days since admission. Vascular risk factors of cases were collected according to the description of patients. Sysmex XE-2100 automatic blood cell analyzer and related reagents (Sysmex) were used to determine the WBCs, neutrophils, lymphocytes, RBCs, HB, and PLT. An automated AIA 600 system (Tosho Corporation) was used to determine the TSH, FT3, and FT4.

### Statistical Analysis

All calculations were performed using SPSS version 22.0. All baseline characteristics and risk factors of excluded and included cases were statistically analyzed by logistic regression model and estimate the propensity score. Their distributions were also compared using the Mann–Whitney *U*-test or the chi-square test. For baseline characteristics and risk factors of included cases, categorical variables were compared using the chi-square test, and continuous variables were compared using the Mann–Whitney *U* test among the different groups. *p* < 0.05 was considered statistically significant. Univariate logistic regression was used for identifying risk factors, and factors with *p* < 0.1 were analyzed with multivariate logistic regression, in a method of Backward Logistic Regression. The nomograms were constructed using R. After calculating probabilities in multivariate analysis, we assessed the performance of the model by calculating the area under the ROC curve (AUC). The larger the AUC, the more accurate the prediction is. Hosmer–Lemeshow (H–L) goodness of fit test was used to evaluate the calibration degree of models, and models with *p* > 0.1 were considered in good calibration degree.

## Results

**Figure 2:**
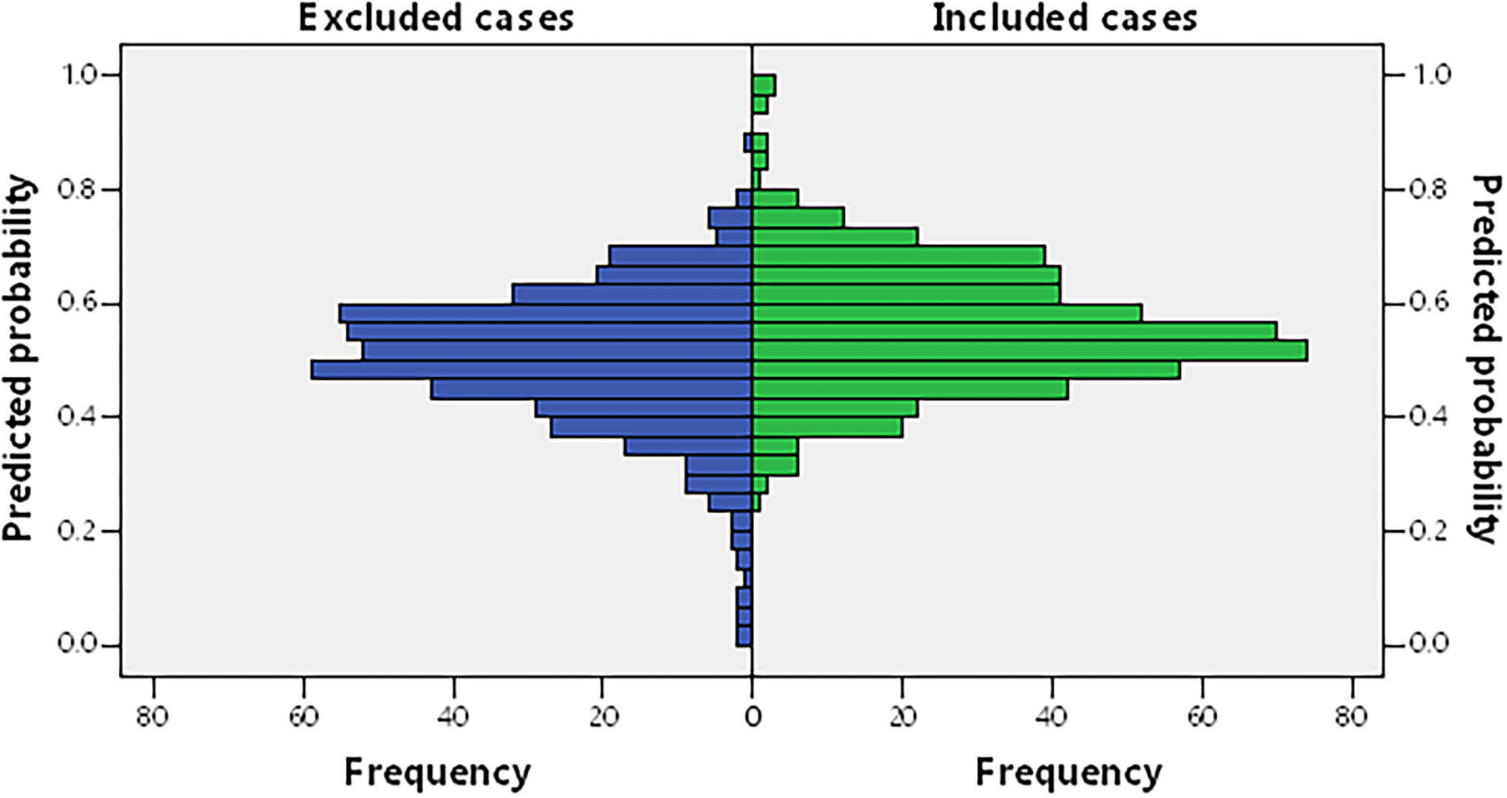
Propensity score of included cases and excluded cases, from demographic and clinical characteristics and the routine serum test. Including age, gender, hypertension, diabetes, coronary artery disease, atrial fibrillation, hyperlipidemia, history of smoking and drinking alcohol, weight, National Institutes of Health Neurological Deficit Score (NIHSS) score at admission and discharge, mean blood pressure (MBP), left ventricular ejection fraction (LVEF), fasting blood glucose (FBG), 2-h post-meal blood glucose (P2hPG), glycated hemoglobin (HbA1c%), total cholesterol (TC), low-density lipoprotein cholesterol (LDL), high-density lipoprotein cholesterol (HDL), cell count of white blood cell (WBC), neutrophil, lymphocyte, red blood cell (RBC), and blood platelet (PLT), hemoglobin (HB), total serum protein (TP), albumin, total prothrombin time (PT), international normalized ratio (INR), activated partial thromboplastin time (APTT), fibrinogen (FIB), urea nitrogen (BUN), creatinine (Cr), homocysteine (HCY), thyroid-stimulating hormone (TSH), free triiodothyronine (FT3), and free tetraiodothyronine (FT4).

**Figure 3:**
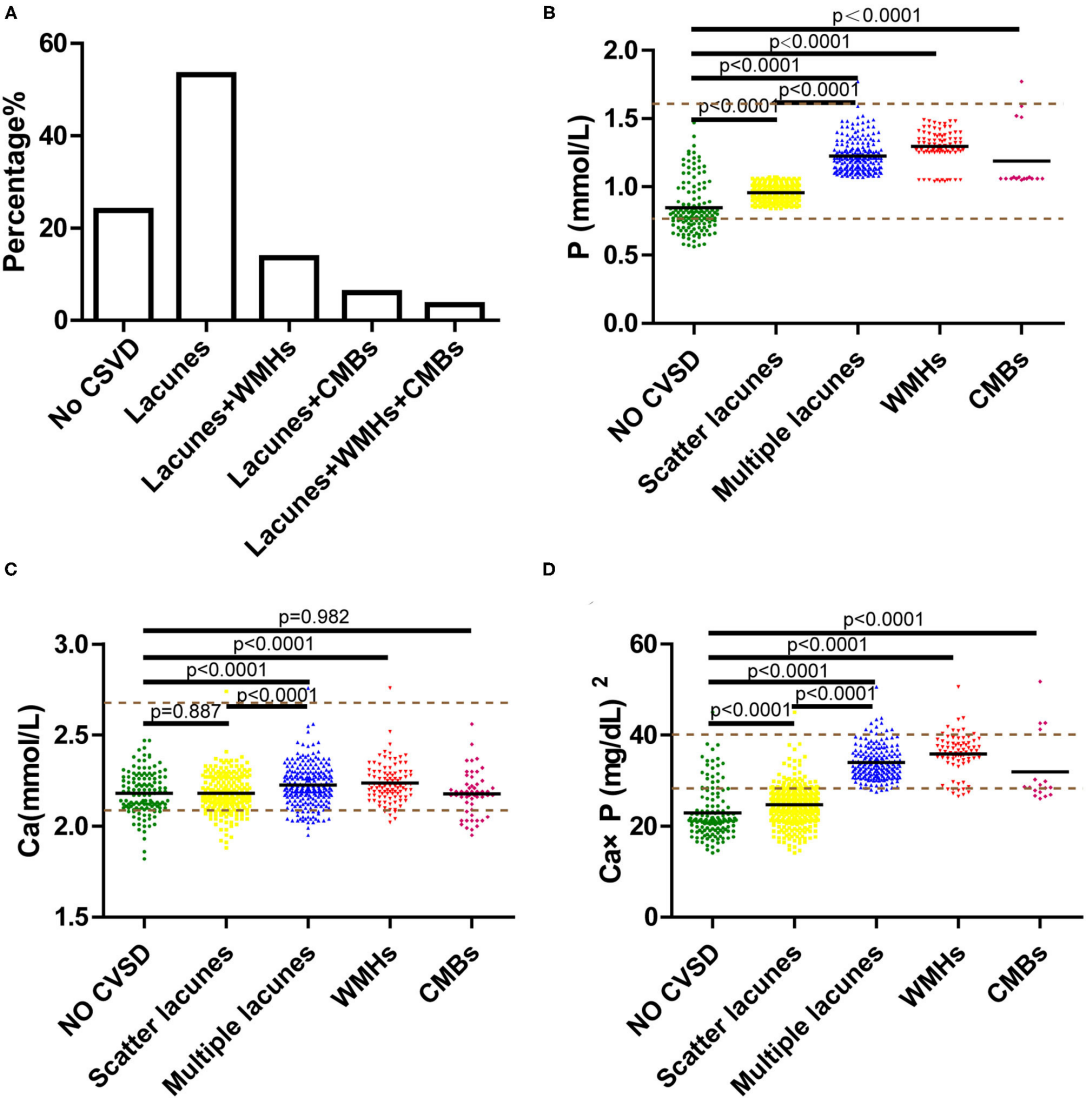
**(A)** Percentage of single or combined subtypes in cerebral small vessel disease (CSVD). **(B–D)** Serum phosphorus (P), calcium (Ca), calcium-phosphate product (Ca × P) of subtypes in CSVD, including scattered lacunes, multiple lacunes, white matter hyperintensities (WMHs), and cerebral microbleeds (CMBs). Brown dotted lines are the upper and lower limits of normal values.

**Figure 4:**
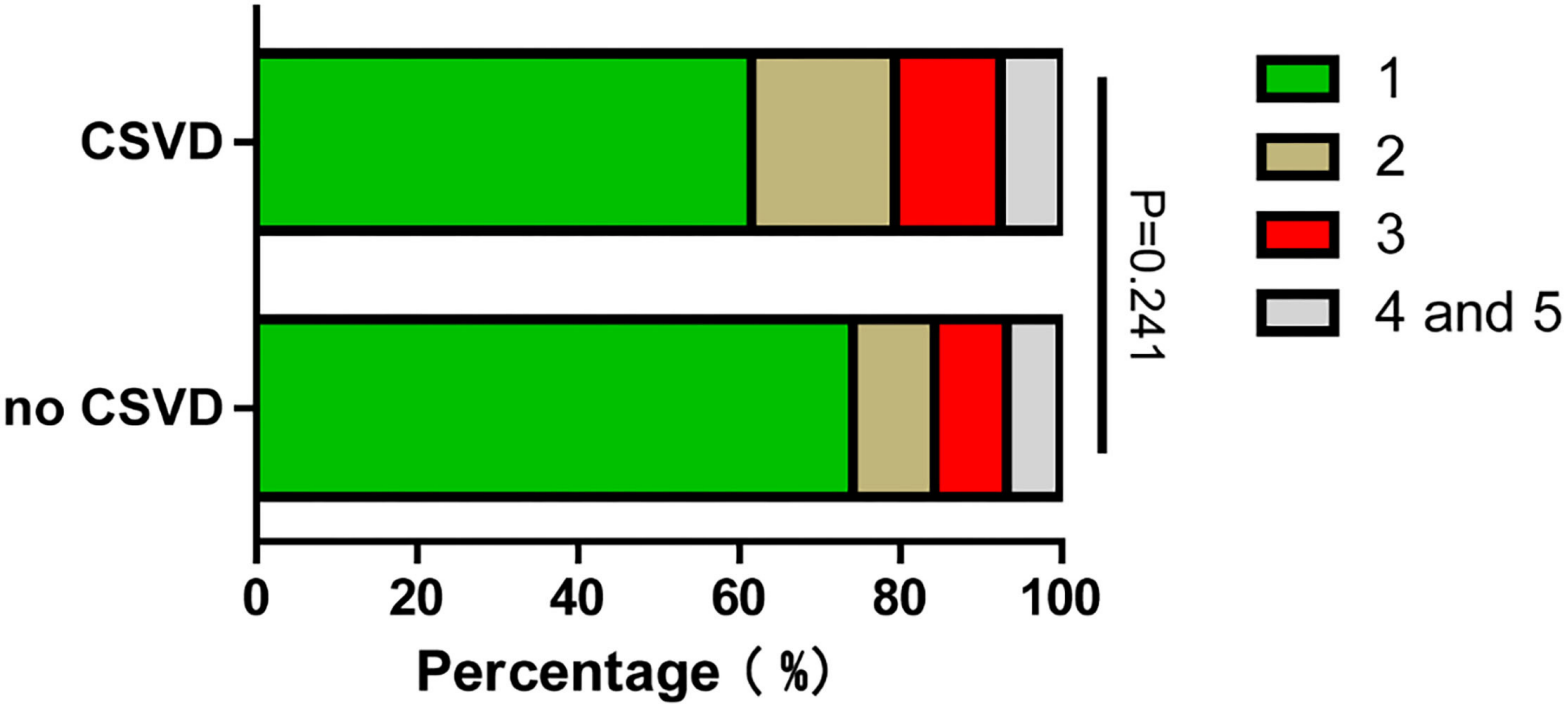
TOAST classification distribution of no CSVD and CSVD groups. TOAST classification: (1) Large artery atherosclerosis. (2) Cardioembolism. (3) Small-artery occlusion. (4) Stroke of other determined cause. (5) Stroke of undermined cause.

**Figure 5:**
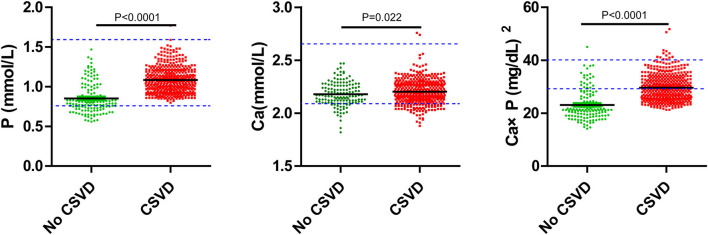
Serum P, Ca, Ca × P, and concentration of no CSVD and CSVD. Blue dotted lines are the upper and lower limits of normal values.

In this study, 588 patients were included and 514 patients were excluded. To check if there was selection bias, we calculated propensity scores of demographic parameters, vascular risk factors, blood biochemistry, and vital signs on admission in excluded and included cases, respectively. Distributions of propensity scores were similar between groups (Figure 2). There were higher rates of coronary artery disease and atrial fibrillation in included cases (Supplementary Table S1). Among included cases, 140 (23.81%) cases were included in the no CSVD group, and 448 cases (76.19%) were included in the CSVD group based on whether presented with signs of CSVD on cranial MR images (Figure 3A). TOAST classification distributions showed no statistical difference among no CSVD and CSVD groups (Figure 4). The general characteristics of the two groups are detailed in Table 1. Age, rate of hypertension, diabetes, CKD2-4, serum P, Ca, Ca × P, and adjusted Ca × P were higher, while the levels of WBC and HB were lower in patients with CSVD than patients without CSVD (Table 1, Figure 5).

**Table 1:** The general characteristics of patients without cerebral small vessel disease (CSVD) and with CSVD.

**Baseline characteristics**	**No CSVD**	**CSVD**	***P*** **-value**
N	140	448	
Age, years	58.82 ± 12.99	70 (62, 77)	<0.001
Female	45 (32.14%)	155 (34.59%)	0.661
Hypertension	100 (71.43%)	393 (88.51%)	<0.001
Diabetes	39 (27.86%)	181 (40.58%)	0.007
Coronary artery disease	18 (13.95%)	76 (18%)	0.349
Atrial fibrillation	19 (13.57%)	68 (15.21%)	0.685
Hyperlipidemia	69 (49.64%)	216 (48.98%)	0.923
History of smoking	69 (49.29%)	191 (42.73%)	0.174
Drinking alcohol	54 (38.57%)	147 (32.89%)	0.222
Weight, kg	65 (60, 69.8)	65 (59, 70)	0.369
NIHSS score	2 (1, 4)	2.5 (1, 4)	0.864
MBP, mmHg	104.04 ± 14.63	106.67 (97, 12.67)	0.082
FBG, mmol/L	5.15 (4.5, 6.55)	5.2 (4.5, 6.65)	0.655
P2hPG, mmol/L	8.58 (6.63, 11.84)	8.18 (6.37, 11.3)	0.281
HbA1c, %	6.1 (5.6, 7.18)	6.1 (5.6, 7)	0.590
TC, mmol/L	4.51 (3.82, 5.08)	4.53 (3.79, 5.32)	0.612
TG, mmol/L	1.6 (1.145, 2.23)	1.5 (1.14, 2.06)	0.146
LDL, mmol/L	2.55 (2.07, 3.07)	2.57 (2.08, 3.2)	0.521
HDL, mmol/L	0.99 (0.84, 1.178)	1.04 (0.88, 1.22)	0.117
LVEF, %	65 (61.5, 68.72)	65.3 (61.3, 68.6)	0.984
WBC, × 10^9^/L	7.01 (5.66, 8.55)	6.54 (5.43, 7.82)	0.023
Neutrophil, × 10^9^/L	4.34 (3.365, 5.64)	4.02 (3.15, 5.33)	0.080
Lymphocyte, × 10^9^/L	1.7 (1.32, 2.18)	1.69 (1.31, 2.05)	0.529
RBC, × 10^12^/L	4.55 (4.24, 4.83)	4.5 (4.11, 4.83)	0.080
HB, g/L	139 (128.25, 148.75)	135 (124, 146)	0.032
TP, g/L	67.25 (63.25, 69.75)	67.1 (63.7, 70.2)	0.465
Albumin, g/L	37.75 (34.8, 39.58)	38.1 (35.6, 39.9)	0.329
PLT, × 10^9^/L	218 (172, 250)	224 (184, 262)	0.135
PT, s	13.5 (13.1, 14.28)	13.5 (13, 14)	0.367
INR	1.04 (0.99, 1.11)	1.03 (0.98, 1.09)	0.483
APTT, s	36.9 (34.03, 39.58)	37.1 (34.9, 39.6)	0.358
FIB, g/L	3.48 (2.98, 4.22)	3.63 (2.96, 4.41)	0.271
BUN, mmol /L	4.8 (3.8, 6)	5 (4.1, 6.2)	0.083
Cr, μmol /L	71 (60, 83.75)	68 (58, 82)	0.599
CKD			0.001
1(eGFR ≥ 90)	69(49.29%)	136(30.36%)	
2(60 ≤ eGFR <90)	48(34.29%)	192(42.86%)	
3(30 ≤ eGFR <60)	22(15.71%)	113(25.22%)	
4(10 ≤ eGFR <30)	1(0.71%)	7(1.56%)	
HCY, μg/L	10 (8, 12)	10 (8, 13)	0.055
TSH, mIU/L	1.46 (0.99, 2.39)	1.62 (1.05, 2.39)	0.563
FT3, pmol//L	4.4 (4, 4.78)	4.5 (4.1, 4.8)	0.235
FT4, pmol//L	11.23 (10.21, 12.79)	11.23 (10.14, 12.62)	0.588
*P*, mmol/L	0.81 (0.73, 0.98)	1.06 (0.95, 1.19)	<0.001
Ca, mmol/L	2.18 ± 0.11	2.2 (2.13, 2.28)	0.022
Adjusted Ca^*^, mmol/L	2.22 ± 0.10	2.23 (2.17, 2.29)	0.135
Ca × P, (mg/dL)^2^	21.36 (19.1, 26.29)	28.94 (25.64, 32.98)	<0.001
Adjusted Ca × P, (mg/dL)^2^	21.84 (20.09, 26.29)	29.22 (25.98, 33.28)	<0.001

*Normally distributed data are represented by mean ± SD, skewed distribution data are represented by median (IQR), and frequency data are represented by n (%)*.

* *Adjusted calcium (Ca) (mmol/L) = Ca (mmol/L) + 0.02 [40 – albumin (g/L)], serum Ca is adjusted if albumin < 35 g/L, or >51 g/L. For male: estimated glomerular filtration rate (eGFR) = (140 – age) × weight (kg) × 1.23/Cr (μmol/L); for female: eGFR = (140 – age) × weight (kg) × 1.03/Cr (μmol/L). Factors with p < 0.05 are statistically significant*.

**Figure 6:**
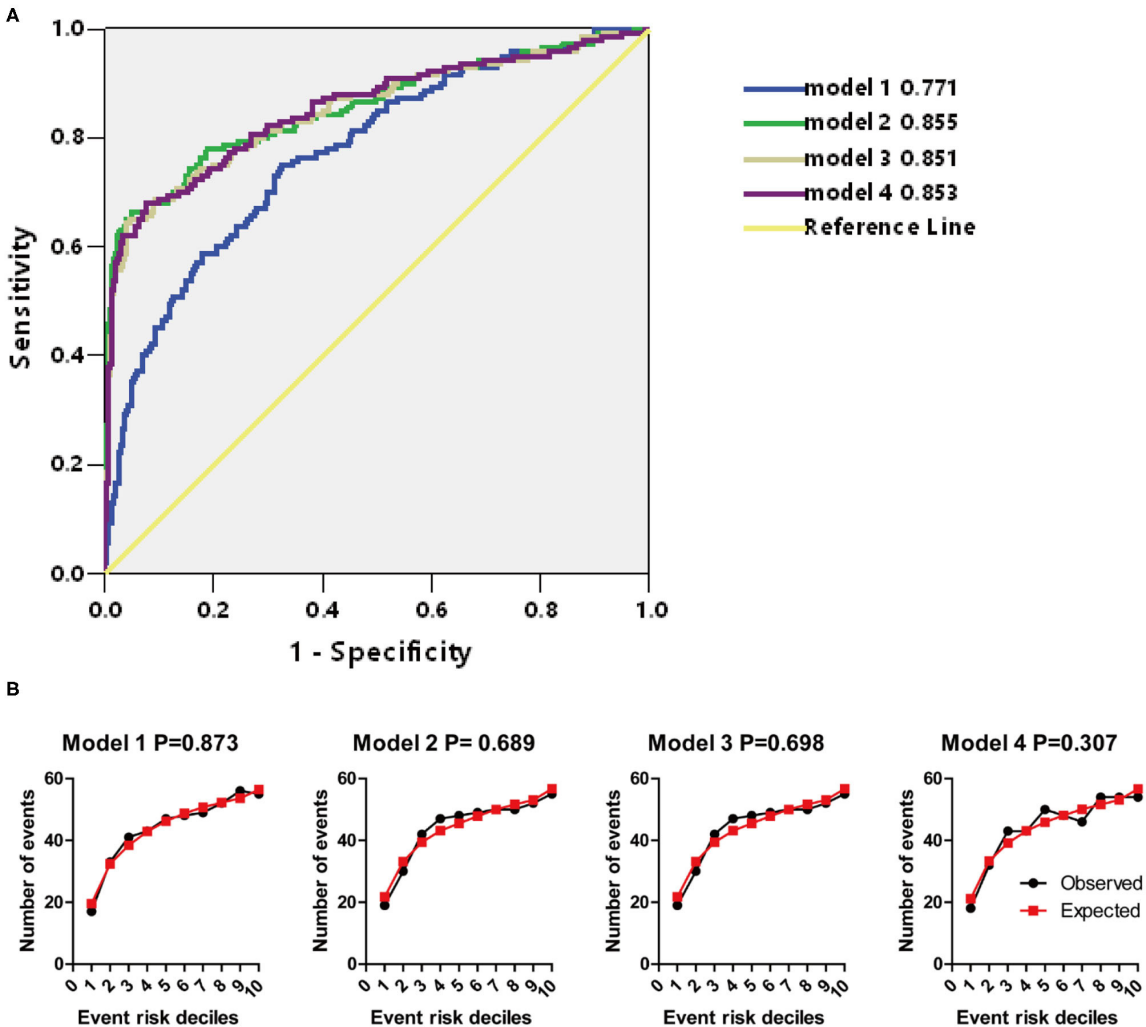
**(A)** AUCs of models 1–4. **(B)** Hosmer–Lemeshow (H–L) test of models 1–4.

Univariate and multivariate logistic regressions were used to identify potential risk factors of CSVD and further construct models to predict the risk of CSVD. Receiver operating characteristic (ROC) curves were used as the discrimination index of models. H–L test was used to evaluate the calibration degree of models. All factors with a *p* < 0.2 in Table 1 were analyzed by univariate logistic regression (Table 2). The OR of P was extremely high, we tried to transform the data by 1/P or log10(P + 1), to improve the normality, but the B and OR of P were still not improved, and we speculated it was caused by the unbalanced distribution of P between no CSVD and CSVD cases (Supplementary Table S2). Baseline factors with *p* < 0.1 in Table 2, including age, hypertension, diabetes, MBP, WBC, RBC, HB, BUN, and CKD, were analyzed with multivariate logistic regression, factors with *p* < 0.05, including age, hypertension, diabetes, MBP, WBC, BUN, were included in model 1 ultimately (Table 3), AUCs is 0.771, and *p*-value of H–L test is 0.873 > 0.05 (Figures 6A,B). RBC and HB were highly correlated, but they were excluded in model 1 (Supplementary Table S3). CKD seemed to be a more sensitive indicator of renal function, but it was also excluded in model 1, and it was moderately correlated with age and weakly correlated with a BUN (Supplementary Table S3). Among the included variables, age and MBP were weakly correlated with hypertension, so the variables were relatively independent of each other.

**Table 2:** Univariate analysis for factors associated with CSVD by binary regression.

**Baseline characteristics**	**OR**	**95%CI**	***P*** **-value**
**N 140/448**			
Age, years	1.077	1.058–1.097	<0.001
Hypertension	3.090	1.934–4.937	<0.001
Diabetes	1.769	1.168–2.679	0.007
History of smoking	0.772	0.527–1.129	0.182
MBP, mmHg	1.015	1.002–1.029	0.027
HDL, mmol/L	1.566	0.796–3.080	0.194
WBC, × 10^9^/L	0.930	0.857–1.010	0.086
Neutrophil, × 10^9^/L	0.941	0.862–1.029	0.182
RBC, × 10^12^/L	0.714	0.512–0.995	0.047
HB, g/L	0.988	0.977–0.999	0.039
PLT, × 10^9^/L	1.002	0.999–1.005	0.114
BUN, mmol /L	1.090	0.993–1.196	0.069
CKD2(60 ≤ eGFR <90); CKD1 as ref	2.029	1.322–3.116	0.001
CKD3(30 ≤ eGFR <60); CKD1 as ref	2.606	1.517–4.476	<0.001
CKD4(10 ≤ eGFR <30); CKD1 as ref	3.044	0.359–25.789	0.307
P, mmol/L	8,473.940	1,522.947–47,150.476	<0.001
Ca, mmol/L	5.190	1.025–26.290	0.047
Adjusted Ca, mmol/L	3.118	0.514–18.928	0.217
Ca × P, (mg/dL)^2^	1.335	1.262–1.412	<0.001
Adjusted Ca × P, (mg/dL)^2^	1.345	1.269–1.425	<0.001

*Factors with p < 0.2 are listed*.

**Table 3:** Multivariate analysis for models by binary logistic regression.

	**Model 1**				**Model 2**				**Model 3**				**Model 4**		
	***P*** **-value**	**OR**	**95%CI**		***P*** **-value**	**OR**	**95%CI**		***P*** **-value**	**OR**	**95%CI**		***P*** **-value**	**OR**	**95%CI**
Age	<0.001	1.080	1.059–1.101	Age	<0.001	1.049	1.027–1.071	Age	<0.001	1.048	1.026–1.070	Age	<0.001	1.048	1.027–1.070
Hypertension	0.030	1.896	1.063–3.384	Hypertension	0.002	2.745	1.470–5.127	Hypertension	0.001	2.950	1.598–5.447	Hypertension	0.001	2.863	1.551–5.286
Diabetes	0.016	1.753	1.110–2.767	Diabetes	NE			Diabetes	NE			Diabetes	NE		
MBP	0.013	1.021	1.004–1.037	MBP	NE			MBP	NE			MBP	NE		
WBC	0.012	0.889	0.811–0.974	WBC	NE			WBC	NE			WBC	NE		
BUN	0.049	1.114	1.001–1.240	BUN	NE			BUN	NE			BUN	NE		
				P	<0.001	3,720.401	646.665–21,404.249	Ca × P,	<0.001	1.294	1.222–1.370	Adjusted Ca × P,	<0.001	1.302	1.228–1.381
				Ca or adjusted Ca	NE										

*Model 1: adjusted for covariates that p < 0.1 in univariate analysis. Model 2 = model 1 + phosphorus (P), Ca, or adjusted Ca. Model 3 = model 1 + Ca × P. Model 4 = model 1 + adjusted Ca × P. A method of backward logistic regression is used, and factors with p > 0.05 are removed from models. NE, not entry*.

**Figure 7:**
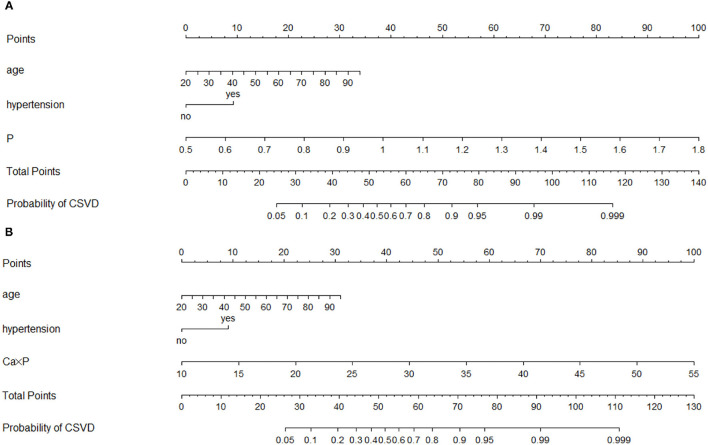
Nomograms of models 2 and 3 for predicting the probability of CSVD. **(A)** Model 2; **(B)** Model 3.

Based on model 1, we constructed model 2 (including P, Ca, or adjusted Ca), model 3 (including Ca × P), and model 4 (including adjusted Ca × P) (Table 3). In these three models, only age and hypertension were remained in the end. P was included in model 2, Ca × P was included in model 3, and adjusted Ca × P was included in model 4 (Table 3). Among the included variables, age was weakly correlated with P, Ca × P, and adjusted Ca × P (Supplementary Table S3). AUCs were similar among model 2 to model 4 (0.855, 0.851, 0.853, respectively), which are higher than model 1 (Figure 6A). *p*-values of H–L test among these 3 models are 0.689, 0.698, and 0.307, respectively (Figure 6B). The calibration degree of model 4 was worse than those in models 2 and 3, thus we did not include adjusted Ca × P in the prediction model. Instead, we included P, Ca, or adjusted Ca, and Ca × P to establish nomograms to predict the probability of CSVD (Figure 7). Age, hypertension, P, or Ca × P were all positively associated with CSVD. The OR of P was 3,720.401 (646.665–21,404.249), and OR of Ca × P was (1.294, 1.222–1.370) (Table 3). Thus, P and Ca × P are potential CSVD risk factors.

**Figure 8:**
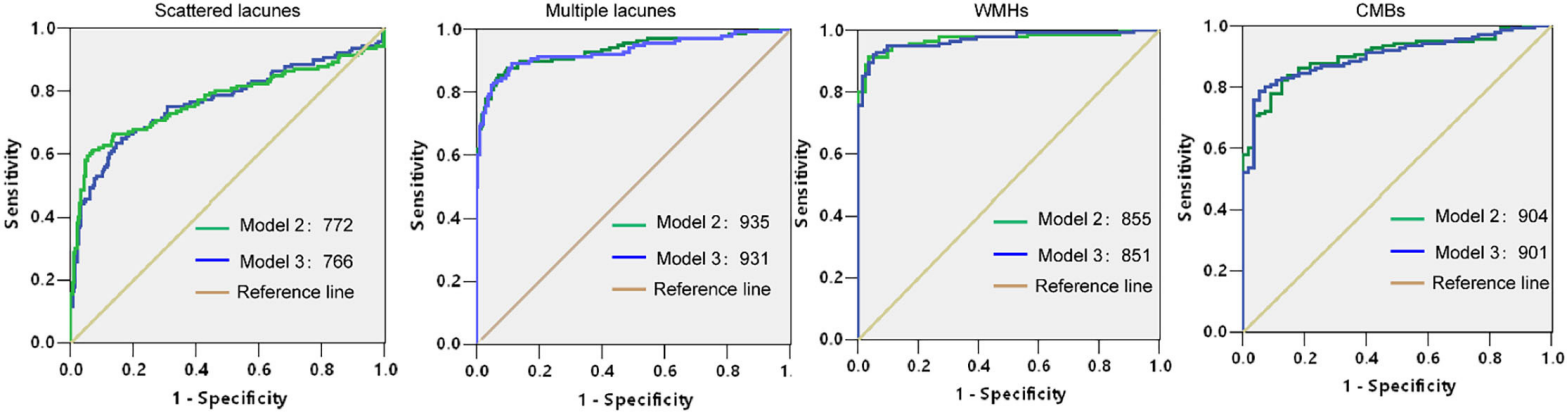
Receiver operating characteristics (ROCs) and AUCs of models 2 and 3 in subtypes of CSVD.

We explored the incidence of subtypes of CSVD, including lacunes, WMHs, and CMBs, and patients with lacunes were further divided into scattered lacunes and multiple lacunes. Then, we validated if models 2 and 3 were valid in these subtypes. We found that lacunes were the most common types in CSVD, and 22.96% of the patients with CSVD have two or three types coexisted (Figure 3A). P and Ca × P were significantly increased in subgroups of CSVD than no CSVD group and were highest in multiple lacunes groups. Ca is slightly higher in the multiple lacunes and WMH group than in the no CSVD group (Figures 3B–D). P and Ca × P remained to be risk factors in each subgroup of CSVD (Tables 4–6). AUCs and H–L test show good discrimination and calibration power of models in each subgroup (Figure 8, Table 7).

**Table 4:** Multivariate analysis for factors associated with lacunes by multinominal regression.

	**Model 2**				**Model 3**		
	***P*** **-value**	**OR**	**95%CI**		***P*** **-value**	**OR**	**95%CI**
**Scattered lacunes (*****n*** **=** **212, no CVSD as ref)**								
Age	<0.001	1.051	1.027–1.075	Age	<0.001	1.050	1.027–1.074
Hypertension	0.024	2.281	1.116–4.662	Hypertension	0.018	2.350	1.159–4.766
Diabetes	0.128	1.497	0.891–2.517	Diabetes	0.121	1.501	0.898–2.510
MBP	0.161	1.014	0.995–1.033	MBP	0.129	1.015	0.996–1.034
WBC	0.036	0.883	0.786–0.992	WBC	0.020	0.874	0.780–0.979
BUN	0.100	1.118	0.979–1.276	BUN	0.084	1.121	0.985–1.275
P	<0.001	161.836	22.407–1,168.871	Ca × P	<0.001	1.159	1.086–1.237
Ca or adjusted Ca	0.592	0.547	0.060–4.982				
**Multiple lacunes (*****n*** **=** **236, no CVSD as ref)**								
Age	0.000	1.078	1.043–1.113	Age	0.000	1.081	1.047–1.115
Hypertension	0.071	2.547	0.922–7.052	Hypertension	0.036	2.894	1.070–7.824
Diabetes	0.685	1.153	0.577–2.298	Diabetes	0.588	1.204	0.616–2.352
MBP	0.017	1.031	1.005–1.057	MBP	0.013	1.031	1.007–1.057
WBC	0.602	0.962	0.832–1.112	WBC	0.413	0.945	0.824–1.083
BUN	0.019	1.210	1.031–1.420	BUN	0.016	1.213	1.036–1.420
P	0.000	945,018,125.529	36,026,472.282	Ca × P	0.000	1.871	1.687–2.074
Ca or adjusted Ca	0.768	1.593	0.071–34.941				

*Factors with p < 0.05 entered into models*.

**Table 5:** Multivariate analysis for factors associated with white matter hyperintensities (WMHs) by binary regression.

	**Model 2**				**Model 3**		
	***P*** **-value**	**OR**	**95%CI**		***P*** **-value**	**OR**	**95%CI**
Age	<0.001	1.122	1.071–1.176	Age	<0.001	1.118	1.068–1.171
Hypertension	NE			Hypertension	0.039	3.767	1.068–13.284
Diabetes	NE			Diabetes	NE		
MBP	NE			MBP	NE		
WBC	NE			WBC	NE		
BUN	0.036	1.256	1.014–1.555	BUN	0.023	1.298	1.036–1.625
P	<0.001	6,965.965	576.828–84,123.332	Ca × P	<0.001	1.338	1.231–1.454
Ca or adjusted Ca	NE						

*Method: backward logistic regression. N = 100 in WMHs. NE, no entry*.

**Table 6:** Multivariate analysis for factors associated with cerebral microbleeds (CMBs) by binary regression.

	**Model 2**				**Model 3**		
	***P*** **-value**	**OR**	**95%CI**		***P*** **-value**	**OR**	**95%CI**
Age	0.004	1.057	1.017–1.097	Age	0.001	1.068	1.027–1.121
Hypertension	NE			Hypertension	NE		
Diabetes	NE			Diabetes	NE		
MBP	NE			MBP	NE		
WBC	NE			WBC	NE		
BUN	NE			BUN	0.047	1.248	1.003–1.552
P	<0.001	558.429	49.395–6,313.236	Ca × P	<0.001	1.213	1.121–1.312
Ca or adjusted Ca	NE						

*Method: backward logistic regression. N = 55 in CMBs. NE, no entry*.

**Table 7:** Hosmer–Lemeshow (H–L) test of models 2 and 3 in subtypes of CSVD.

	**Scattered lacunes**	**Multiple lacunes**	**WMHs**	**CMBs**
Model 2	0.858	0.404	0.693	0.541
Model 3	0.571	0.404	0.295	0.560

*Models with p > 0.1 means good calibration power*.

## Discussion

Using a hospital stroke registry of the First Affiliated Hospital of Wenzhou Medical, we found that 76.19% of stroke patients with relatively normal kidney function were concomitant with CSVD. Higher serum P and Ca × P levels were positively linked to CSVD and subtypes of CSVD, including lacunes, WMHs, and CMBs. The OR of P was extremely high in model 2, and we speculated it was caused by the unbalanced distribution of P and relatively small sample size since the OR of Ca × P in model 3 may be more convincing. High P and Ca could promote large vessel calcification, and their influences can mutually be re-enforcing (8–10). CSVD is concomitant with stroke in South China, indicating that they may share some common risk factors or pathologies, although there are two different kinds of diseases. Our results indicate that higher serum P and Ca × P may promote CSVD even when they are at normal levels. Studies of CSVD and CPM have some discrepancies in different regions. A study in Taiwan shows that circulatory P levels > 3.925 mg/dl were associated with severe WMHs in a community-based longitudinal aging study (11). A study in Turkey that follows subjects who underwent brain imaging for any reason reported that patients with CSVD had lower P than normal subjects (12). In the US veteran population, high serum P is associated with CSVD and dementia, and this relationship is more significant in participants <60 years old (13). CPM is significantly affected by light and diet in the normal population, and further large-scale cohort studies on the relationship of CPM and CSVD tracking of the localized community are needed.

Calcium and phosphorus metabolism disorders are common in chronic kidney disease, and patients with CKD have increased CSVD prevalence. Most CPM-related clinical investigations are conducted in the chronic kidney disease population and take large blood vessel calcification as a research target (6, 14). In this study, CKD did not enter to models, we speculated that it was because most cases were in normal kidney function or mild kidney failure, and the small size of patients with CKD 3–5 reduced the sensitivity of models to CKD. The ratios of CKD2 and CKD3 in CSVD cases were still higher than those in no CSVD cases. The contribution of CKD to CSVD should be studied in populations with no selection bias for renal function.

The primary underlying initiating cause of CSVD is the derangement of the blood-brain barrier (BBB), and this may start some years before the first symptoms, leading to the small vessel structural changes and perivascular changes (15). The cellular structure of the BBB is mainly composed of brain microvascular endothelial cells (MVECs) (16). Junction protein (zona occludens-1, occludin, and claudin-5) expression is downregulated in human brain MVECs after high phosphorus treatment (11). Many BBB dysfunction diseases are associated with increasing intracellular Ca levels of MVECs and serum Ca. Many cell membrane ion channels are responsible for regulating intracellular Ca balance. Plasma membrane calcium ATPase could export calcium, and calcium entry from the extracellular space can occur from store-operated cation channels or receptor-operated cation channels (17). In addition, voltage-gated L-type calcium channels and the transient receptor potential superfamily have been identified to comprise calcium influx channels in endothelial cells (18).

High levels of phosphate accelerate the precipitation of calcium and phosphate in the form of hydroxyapatite, accelerating vascular calcification (19). Calcification is associated independently with high calcium levels and is synergistic with elevated phosphate levels (20, 21). High phosphate can directly induce phenotypic transformation of vascular smooth muscle cells (VSMCs) into osteoblasts by stimulating VSMCs to express core-binding factor-a1, repress the production of calcification inhibitors, and promote the release of extracellular vesicles (EVs) lacking these inhibitors, but rich in pro-calcific proteins, such as tissue-nonspecific alkaline phosphatase (22). miR-29b, miR-133b, and miR-211 have direct roles in the vascular smooth muscle calcification induced by high phosphorus (9). In VSMCs, the sodium-phosphorus cotransporter PiT-1 promotes matrix calcification caused by elevated phosphorus, while PiT-2 inhibits its changes (23). Vitamin K-dependent matrix Gla protein (MGP) is a key inhibitor in the formation of vascular calcification, and elevated phosphate and calcium levels increased MGP levels, as an inherent protective mechanism of vascular calcification (24).

The Chinese diet had lower daily intakes of Ca and P, compared with the Japanese, American, and Italian diets (25). Southern Chinese had a relatively higher intake of Ca and P than Northern Chinese, and they were associated with north-south blood pressure differences (26). Serum Ca and P are closely related to CPM, and bones are the largest reserve pool; therefore, although low serum P and Ca × P were a protective factor of CSVD in our study, this conclusion could not be generalized to dietary Ca and P intake. The relationship of dietary Ca, P intake, and CSVD needs further research.

Although this study showed propensity score distribution of the included and excluded data to confirm that the baseline characteristic of the excluded cases was similar to that of the included cases. However, the analyzed data were from a single-center medical institution in Southern China. The population in the hospital had higher health literacy, compared with those who did not seek medical attention; the selected population had geographic and ethnic specificities; the awareness rate of vascular risk factors varied. All the above factors lead to an inevitable selection bias and recalling bias of the study. Further multicenter, multiethnic, and community-based research should be conducted. There were some limitations of real-world research, for example, body mass index (BMI) cannot be calculated and counted because there were too many missing height data, weight was counted instead, and this led to inadequate assessment of obesity in this study.

In conclusion, serum P and Ca × P were higher in patients with CSVD, including subtypes of lacunes, especially multiple lacunes, WMHs, and CMBs. Patients with elder age, hypertension, higher P, or Ca × P had an increased risk of CSVD in patients with stroke who did not have CPM disorder. Higher P or Ca × P are also risk factors of CSVD subtypes, including lacunes, WMHs, and CMBs.

## Data Availability Statement

The raw data supporting the conclusions of this article will be made available by the authors, without undue reservation.

## Ethics Statement

The studies involving human participants were reviewed and the Ethical Decision Committee of the Research Administration at First Affiliated Hospital of Wenzhou Medical University approved the study. The patients/participants provided their written informed consent to participate in this study.

## Author Contributions

WL and ZW were responsible for data statistics and writing the manuscript. CC and JJ collected data. BD provided resources and designed the study. All authors contributed to the article and approved the submitted version.

## Funding

This study was supported by the National Natural Science Foundation of China (Grant No. 81901273) and the Science Technology Department of Zhejiang Province (Grant No. Q21H090076).

## Conflict of Interest

The authors declare that the research was conducted in the absence of any commercial or financial relationships that could be construed as a potential conflict of interest.

## Publisher's Note

All claims expressed in this article are solely those of the authors and do not necessarily represent those of their affiliated organizations, or those of the publisher, the editors and the reviewers. Any product that may be evaluated in this article, or claim that may be made by its manufacturer, is not guaranteed or endorsed by the publisher.

## Abbreviations

MBP: Mean blood pressure
LVEF: left ventricular ejection fraction
FBG: fasting blood glucose
P2hPG: 2-h post-meal blood glucose
HbA1c%: glycated hemoglobin
TC: total cholesterol
TG: triglyceride
LDL: low-density lipoprotein cholesterol
HDL: high-density lipoprotein cholesterol
WBC, white blood cell;, RBC: red blood cell
PLT: blood platelet
HB: hemoglobin
TP: total serum protein
PT: total prothrombin time
INR: international normalized ratio
APTT: activated partial thromboplastin time
FIB: fibrinogen
BUN: urea nitrogen
Cr: creatinine
HCY: homocysteine
TSH: thyroid-stimulating hormone
FT3: free triiodothyronine
FT4: free tetraiodothyronine
NIHSS: National Institutes of Health Neurological Deficit Score
CSVD: cerebral small vascular disease
WMHs: white matter hyperintensities
CMBs, cerebral microbleeds, CPM: calcium and phosphorus metabolism
CKD: chronic kidney diseases
MVECs: microvascular endothelial cells
VSMCs: vessel smooth muscular cells
EVs: extracellular vesicles
MGP: matrix Gla protein
CKD: chronic kidney diseases.
