# Supplementary material for: A High Serum Phosphate and Calcium-Phosphate Product Is Associated With Cerebral Small Vascular Disease in Patients With Stroke: A Real-World Study
Source: Front Nutr. 2022 Apr 4;9:801667. doi: 10.3389/fnut.2022.801667 (PMC9013770; doi:10.3389/fnut.2022.801667)
Supplement: Supplementary file 1 [file Data_Sheet_1.docx]

Table S1 The general characteristics of included and excluded patients.

| Baseline characteristics | Included cases | Excluded cases | P-value |
| --- | --- | --- | --- |
| N | 588 | 514 |  |
| Age, years | 67(58,76) | 67(58,75) | 0.496 |
| Female | 33.8% | 31.6% | 0.439 |
| Hypertension | 84.5% | 84.9% | 0.866 |
| Diabetes | 38.9% | 40.7% | 0.577 |
| Coronary artery disease | 16.7% | 11.1% | 0.012 |
| Atrial fibrillation | 14.7% | 9.3% | 0.007 |
| Hyperlipidemia | 49.4% | 49.6% | 0.951 |
| History of smoking | 44.6% | 47.1% | 0.429 |
| Drinking alcohol | 34.4% | 36.1% | 0.569 |
| Weight, kg | 64.42(59.20, 69.50) | 64.0(057.58, 70.00) | 0.343 |
| NIHSS score | 3(2, 5) | 4(2, 6) | 0.063 |
| CKD |  |  | 0.779 |
| 1eGFR≥90 | 33.1% | 31.0% |  |
| 260≤eGFR <90 | 43.3% | 43.9% |  |
| 330≤eGFR <60 | 22.6% | 23.9% |  |
| 410≤eGFR <30 | 1.02% | 1.19% |  |

Skewed distribution data is represented by median IQR, and frequency data is represented by n %. Factors with P<0.05 is statistically significant.

Table S2. Characteristic analysis of P, 1/P and log10(P+1), and univariate analysis with CSVD by Binary Regression

| Data | Group | Characteristic of data | | | Binary Regression | | | |
| --- | --- | --- | --- | --- | --- | --- | --- | --- |
|  |  | Kurtosis | Skewness | P-value of Normality | B | Constant | OR | P-value of Regression |
| P, (mmol/L) | No CSVD | .603 | 1.075 | ＜0.001 | 9.708 | -8.174 | 8473.940 | 8.24e-026 |
|  | CSVD | .107 | .818 | ＜0.001 |  |  |  |  |
| 1/P | No CSVD | -.439 | -.103 | .028 | -8.875 | 10.622 | 1.39 e-4 | 5.05e-027 |
|  | CSVD | -.782 | -.076 | ＜0.001 |  |  |  |  |
| Log10(P+1) | No CSVD | -.013 | .748 | ＜0.001 | 41.90 | -11.035 | 1.58e+018 | 6.13e-026 |
|  | CSVD | -.217 | .507 | ＜0.001 |  |  |  |  |

|  | Age | Hypertension | Diabetes | MBP | WBC | RBC | HB | BUN | CKD | P | Ca | Ca×P | Adjusted Ca×P |
| --- | --- | --- | --- | --- | --- | --- | --- | --- | --- | --- | --- | --- | --- |
| Age | 1 | .107 | - | - | - | - | - | - | .444 | .330 | .091 | .336 | .334 |
| Hypertension | .107 | 1 |  | .340 |  |  |  | - | - |  |  |  |  |
| Diabetes | - |  | 1 |  |  |  |  | - | - | .093 | - | .082 | - |
| MBP | - | .340 |  | 1 |  |  |  |  |  |  |  |  |  |
| WBC | - |  |  |  | 1 |  |  |  |  |  |  |  |  |
| RBC | - |  |  |  |  | 1 | .854 |  |  |  |  |  |  |
| HB | - |  |  |  |  | .854 | 1 |  |  |  |  |  |  |
| BUN | - |  |  |  |  |  |  | 1 | .359 | .104 | .112 | .129 | .158 |
| CKD | .444 |  |  |  |  |  |  | .359 | 1 | - | - | - | - |
| P | .330 |  | .093 |  |  |  |  | .104 |  | 1 | .176 |  |  |
| Ca | .091 |  | - |  |  |  |  | .112 |  | .176 | 1 |  |  |
| Ca×P | .336 |  | .082 |  |  |  |  | .129 |  |  |  | 1 |  |
| Adjusted Ca×P | .334 |  | - |  |  |  |  | .158 |  |  |  |  | 1 |

Correlation analysis was conducted between independent variables that might have clinical significance, Pearson correlation

coefficients were used if variables were continuous. Spearman correlation coefficients were used if either was rank variable.

Correlation coefficients were listed if p value<0.05. “-” were listed if p value≥0.05. Variables were considered highly correlated

if correlation coefficients≥0.8, moderately correlated if correlation 0.4<coefficients<0.8, weakly correlated if correlation

coefficients≤0.4.

Table S3. Correlation analysis of independent variables
